# Supplementary material for: Insight into the Characterization of Volatile Compounds in Smoke-Flavored Sea Bass (Lateolabrax maculatus) during Processing via HS-SPME-GC-MS and HS-GC-IMS
Source: Foods. 2022 Aug 29;11(17):2614. doi: 10.3390/foods11172614 (PMC9455667; doi:10.3390/foods11172614)
Supplement: Supplementary file 1 [file foods-11-02614-s001.zip › foods-1835081-supplementary.pdf]

Supplementary materials

Table S1. Comparisons of the detected VOCs in the different processing stages by HS-SPME-GC-MS (n = 3).

No.	VOCs	CAS	Retention time	Content (ng/g)				
				Raw	Salted	Dried	Liquid-smoked	Cooked
				**Aldehyde**				
**1**	**Hexanal**	66-25-1	5.718	541.13±37.17	134.37±56.12	441.87±50.28	ND	ND
2	3-Furaldehyde	498-60-2	6.431	ND	ND	ND	7871.42±1005.06	3583.77±356.63
3	Heptanal	111-71-7	9.939	189.79±14.96	39.57±9.75	131.00±51.00	ND	ND
4	Benzaldehyde	100-52-7	12.561	53.54±5.95	ND	60.73±12.35	504.12±175.64	340.92±51.54
5	5-Methyl furfural	620-02-0	12.873	ND	ND	ND	2067.04±913.62	ND
6	Octanal	124-13-0	14.575	132.35±41.13	ND	56.74±11.52	84.80±14.64	54.9±6.10
7	Nonanal	124-19-6	17.694	191.46±34.72	138.94±47.99	140.40±41.53	ND	ND
8	5-Ethyl-2-furaldehyde	23074-10-4	18.384	ND	ND	ND	633.59±55.77	ND
9	Decanal	112-31-2	20.023	9.00±8.61	ND	ND	ND	ND
10	(Z)-7-Hexadecenal	56797-40-1	20.153	ND	ND	30.37±15.30	ND	ND
				**Alcohols**				
1	3-Furanmethanol	4412-91-3	9.06	ND	ND	ND	151.33±34.55	ND
2	1-Methylcyclohexanol	590-67-0	9.51	ND	ND	ND	ND	73.64±4.02
3	2,3-dimethylcyclohexanol	1502-24-5	9.961	ND	ND	ND	ND	255.2±42.10
4	1-Octen-3-ol	3391-86-4	13.672	231.86±55.18	67.18±28.67	147.27±52.00	45.04±13.24	103.29±12.59
5	2-Phenyl-2-norbornanol	16821-80-0	15.103	ND	ND	ND	ND	94.39±1.42
6	4-Ethylcyclohexanol	4534-74-1	15.613	11.61±6.60	ND	ND	ND	ND
7	Geraniol	106-24-1	16.028	ND	ND	ND	ND	87.09±4.52
8	2-Octyn-1-ol	20739-58-6	16.814	67.22±48.84	ND	ND	ND	ND
9	Trans-2-undecen-1-ol	75039-84-8	16.732	ND	ND	19.68±9.73	ND	ND
10	4-Methoxybenzhydrol	720-44-5	17.632	ND	ND	ND	338.77±55.96	ND
				**Ketones**				
1	Hydroxyacetone	116-09-6	4.676	ND	ND	ND	261.02±64.66	145.22±43.33
2	2-Methyl-2-cyclopentene-1-one	1120-73-6	10.137	ND	ND	ND	ND	1576.74±391.68
3	2,3-Dimethylcyclopent-2-en-1-one	1121-05-7	11.965	ND	ND	ND	4588.56±472.47	1017.38±258.99
4	3-Methyl-2-cyclopenten-1-one	2758-18-1	13.378	ND	ND	ND	1524.42±439.92	ND
5	4-Hexen-3-one	2497-21-4	13.775	ND	ND	ND	ND	403.67±30.12
				**Esters**				
1	Methyl acetate	79-20-9	3.899	ND	ND	ND	ND	80.56±7.96
2	2-Methoxycarbonylimidazole	17334-09-7	13.419	ND	ND	ND	ND	139.88±33.07
3	1-octylformate	112-32-3	16.845	ND	ND	ND	ND	449.55±49.18
				**Phenols**				
1	4-Ethylphenol	123-07-9	19.232	ND	ND	ND	944.75±169.62	129.82±46.80
2	Phenol	108-95-2	14.191	ND	ND	ND	6514.69±430.78	ND
3	o-Cresol	95-48-7	16.479	ND	ND	ND	8096.06±927.82	3553.84±1352.06

4	p-Cresol	106-44-5	17.098	ND	ND	ND	5547.21±668.94	1638.42±344.80
5	Guaiacol	90-05-1	17.355	ND	ND	ND	12613.75±3913.38	6202.97±261.64
6	2,4-Dimethylphenol	105-67-9	17.766	ND	ND	ND	7462.02±552.27	ND
7	2-Ethylphenol	90-00-6	18.583	ND	ND	ND	2710.53±244.65	314.85±83.61
8	3,5-Dimethylphenol	108-68-9	18.786	ND	ND	ND	ND	1430.84±540.83
9	2-Methoxy-5-methylphenol	1195-09-1	19.45	ND	ND	ND	6286.64±890.91	2342.08±761.66
10	2,4,6-Trimethylphenol	527-60-6	19.982	ND	ND	ND	894.70±200.01	334.013±53.43
11	4-Propylphenol	645-56-7	21.088	ND	ND	ND	129.27±60.77	ND
12	4-Ethyl-2-methoxyphenol	2785-89-9	21.156	ND	ND	ND	2456.45±263.85	1130.85±757.37
13	2,6-Dimethoxyphenol	91-10-1	22.799	ND	ND	ND	276.59±20.78	245.25±55.19
14	Eugenol	97-53-0	22.917	ND	ND	ND	655.63±33.16	ND
15	Dihydroeugenol	2785-87-7	23.088	ND	ND	ND	174.18±18.69	84.15±5.21
16	Ethylhydroquinone	2349-70-4	18.272	ND	ND	ND	442.56±50.83	ND
				**Furan**				
1	3,4-dimethylfuran	1000458-50-4	10.137	ND	ND	ND	2440.13±550.56	93.44±2.86
2	2-Acetylfuran	1192-62-7	10.423	ND	ND	ND	2863.91±108.86	ND
3	2-Ethyl-5-methyl furan,	1703-52-2	10.485	ND	ND	ND	ND	824.43±54.96
4	5-Methyl-2-acetylfuran	1193-79-9	14.954	ND	ND	ND	209.57±76.05	ND
				**Acids**				
1	2-Ethylcyclohexanol	3760-20-1	16.728	20.18±10.31	ND	ND	ND	ND
2	Palmitoleic acid	373-49-9	30.324	ND	ND	20.95±6.11	ND	ND
3	Palmitic acid	57-10-3	30.469	28.77±9.64	4.78±3.48	64.97±23.67	272.05±104.23	ND
				**Hydrocarbons**				
1	Undecane	1120-21-4	17.576	21.38±8.49	11.65±6.76	184.84±4.25	285.16±4.01	296.44±14.50
2	Dodecane	112-40-3	19.897	46.98±3.07	17.85±11.74	373.26±106.16	441.95±51.27	344.35±44.55
3	Tridecane	629-50-5	21.847	49.80±20.89	12.88±4.74	165.23±17.15	211.25±91.78	161.02±32.46
4	Tetradecane	629-59-4	23.589	ND	ND	27.35±3.42	ND	ND
5	Heptadecane	629-78-7	28.067	23.75±4.52	15.24±3.98	136.41±49.08	106.28±1.38	108.01±67.01
6	Pristane	1921-70-6	28.148	28.49±17.42	14.60±4.17	93.71±7.10	168.99±26.21	162.65±48.97
7	o-Xylene	95-47-6	8.44	ND	ND	ND	ND	137.10±62.30
				**Other compounds**				
1	Isoprocarb	2631-40-5	19.572	ND	ND	ND	363.19±72.89	145.11±33.80
2	4-Ethylanisole	1515-95-3	20.7	ND	ND	ND	1057.55±231.61	486.14±31.11
3	Pyridine	110-86-1	4.373	ND	ND	ND	157.61±24.61	263.10±34.55
4	3,5-Dimethylpyrazole	67-51-6	7.591	ND	ND	ND	453.62±39.88	ND
5	3,4,5-Trimethylpyrazole	5519-42-6	10.512	ND	ND	ND	2885.16±198.99	ND

ND: not detected.

Table S2. Identification of VOCs in smoke-flavoured sea bass by HS-GC-IMS (n = 3).

No.	Compound	CAS#	Molecule Formula	RT[a]	RI[b]	DT[c]
1	1-Hexanol	C111273	$C_6H_{14}O$	865.7	494.723	1.3232
2	3-Methylbutanol	C123513	$C_5H_{12}O$	726.7	253.198	1.2476
3	3-Methylbutanol*	C123513	$C_5H_{12}O$	725.5	251.388	1.4911
4	2-Methyl-1-propanol	C78831	$C_4H_{10}O$	640.2	166.549	1.1794
5	1-Propanol*	C71238	C_3H_8O	580.4	139.304	1.2409
6	1-Propanol	C71238	C_3H_8O	568.3	134.281	1.1133
7	2-Propanol	C67630	C_3H_8O	523.5	115.765	1.1771
8	2-Propanol*	C67630	C_3H_8O	535	120.507	1.2183
9	Heptanal	C111717	$C_7H_{14}O$	898	560.382	1.3329
10	2,4-Heptadienal	C5910850	$C_7H_{10}O$	964.3	716.371	1.6318
11	Benzaldehyde	C100527	C_7H_6O	960	705.541	1.1458
12	Hexanal*	C66251	$C_6H_{12}O$	789.2	357.35	1.5544
13	Hexanal	C66251	$C_6H_{12}O$	789.5	357.84	1.2625
14	Pentanal	C110623	$C_5H_{10}O$	687.9	203.416	1.1861
15	3-Methylbutanal	C590863	$C_5H_{10}O$	672.8	189.29	1.1642
16	3-Methylbutanal*	C590863	$C_5H_{10}O$	658.4	178.092	1.4004
17	Furfural	C98011	$C_5H_4O_2$	828.1	425.222	1.0841
18	Furfural*	C98011	$C_5H_4O_2$	826.9	423.164	1.3309
19	Butanal	C123728	C_4H_8O	595.8	145.68	1.2873
20	2-Methylpropanal	C78842	C_4H_8O	761.3	309.374	1.0887
21	Propanal	C123386	C_3H_6O	507.6	109.158	1.0432
22	3-Pentanone	C96220	$C_5H_{10}O$	976.1	746.324	1.1034
23	2,3-Pentanedione	C600146	$C_5H_8O_2$	689.2	204.739	1.2289
24	3-Hydroxy-2-butanone	C513860	$C_4H_8O_2$	705.6	223.993	1.0616
25	2-Butanone	C78933	C_4H_8O	535.4	120.671	1.0635
26	2,3-Butanedione	C431038	$C_4H_6O_2$	534	120.105	1.1598
27	2-Isobutyl-3-methylpyrazine	C13925069	$C_9H_{14}N_2$	1153.5	1212.297	1.8114
28	2-Ethyl-5-methylpyrazine	C13360640	$C_7H_{10}N_2$	1005.2	821.402	1.6712
29	2-Acetylfuran	C1192627	$C_6H_6O_2$	909.5	585.587	1.1128
30	2-Methylbutyric acid	C116530	$C_5H_{10}O_2$	808.4	390.67	1.4685
31	Ethyl Acetate	C141786	$C_4H_8O_2$	607.9	150.802	1.0924
32	Ethyl Acetate*	C141786	$C_4H_8O_2$	609.1	151.281	1.3351
33	Acetic acid	C64197	$C_2H_4O_2$	582.9	140.322	1.0574

Note: CAS is the registration number of chemical substances by Chemical Abstracts Service. [a] Represents the retention index calculated on MXT-5 column using n-ketones C4-C9 as external standard. [b] Represents the retention time in the capillary GC column. [c] Represents the drift time in the drift tube. * Dimers formed in the IMS drift tube were represented by symbol "*".
